# Identification and Development of KASP Markers for Novel Mutant *BnFAD2* Alleles Associated With Elevated Oleic Acid in *Brassica napus*

**DOI:** 10.3389/fpls.2021.715633

**Published:** 2021-07-26

**Authors:** Ying Fu, Annaliese S. Mason, Yaofeng Zhang, Huasheng Yu

**Affiliations:** ^1^Institute of Crop and Nuclear Technology Utilization, Zhejiang Academy of Agricultural Sciences, Hangzhou, China; ^2^Plant Breeding Department, The University of Bonn, Bonn, Germany

**Keywords:** *Brassica napus*, seed oleic acid, quantitative trait loci, *BnFAD2*, kompetitive allele specific PCR

## Abstract

The fatty acid desaturase *FAD2* genes are the main contributors to oleic acid content, and different *FAD2* alleles can result in different oleic acid contents in rapeseed oil. Hence, identification of allelic variation in *FAD2* is an extremely desirable breeding goal. By performing QTL mapping using 190 F_2:3_ lines genotyped by genome-wide single nucleotide polymorphism (SNP) markers assayed by the *Brassica* 60 K Infinium BeadChip Array, four quantitative trait loci (QTL) for C18:1 content were mapped on chromosomes A01, A05, A09 and C05 over 3 years in a population segregating for oleic acid content. Two *BnFAD2* genes on A05 and C05 were anchored within the QTL intervals, explaining 45–52 and 15–44% of the observed variation for C18:1 content. Sequence polymorphisms between the corresponding coding regions of the parental lines found two single-nucleotide polymorphisms (SNPs) in *BnFAD2.A05* and *BnFAD2.C05*, respectively, which led to the amino acid changes (C421T and G1073E) in the corresponding proteins. The mutation sites of *Bnfad2.A05* and *Bnfad2.C05* alleles were located within the second H-box and near the third H-box motif of the protein, respectively, and were found to be novel mutant alleles. Lines resulting from the combination of these two alleles contained up to 88% oleic acid in their seed oil, compared with 63% in wild-type controls. Two competitive allele-specific PCR (KASP) markers based on these two mutation sites were successfully developed and validated in segregating F_2_ populations. These markers will facilitate breeding for ultra-high seed oleic acid content in oilseed rape.

## Introduction

Oilseed rape (*Brassica napus* L.) is one of the most important oil crops worldwide. The quality of rapeseed oil is mainly determined by the fatty acid composition of the seeds (Napier et al., 2014), in particular by three unsaturated fatty acids: oleic acid (C18:1), linoleic acid (C18:2) and linolenic acid (C18:3) (Micha and Mozaffarian, 2009; Gillingham et al., 2011). High-oleic acid (>75%) oils have several benefits compared to non-high oleic acid oils, including decreasing low-density lipoprotein levels and putatively the risk of cardiovascular disease in humans (Chang and Huang, 1998) as well as superior anti-oxidative ability leading to a longer shelf life of the oil product (Browse et al., 1998; Lauridsen et al., 1999; Przybylski et al., 2013). Currently, the majority of oilseed rape cultivars worldwide are of canola quality (low erucic acid and glucosinolate content) and contain ~55–65% oleic acid content (Long et al., 2018). Identifying novel high oleic acid germplasm to further increase oleic acid content is a major goal for quality breeding of oilseed rape.

To date, several quantitative trait loci (QTL) for oleic acid content have been identified in *Brassica* (Burns et al., 2003; Hu et al., 2006; Zhao et al., 2008; Smooker et al., 2011; Yan et al., 2011; Yang et al., 2012; Wen et al., 2015; Bao et al., 2018; Chen et al., 2018) and association mapping studies (Niklas et al., 2016; Qu et al., 2017; Bao et al., 2018; Zhao et al., 2019). Of these, one QTL on chromosome C01 (Hu et al., 2006) and one major QTL located on A05 (Hu et al., 2006; Yang et al., 2012) were found to contain homologs to Arabidopsis *AtFAD2*, which catalyzes oleic acid (C18:1) into linoleic acid (C18:2), and hence plays essential role in regulating oleic acid content in seeds. In *B. napus*, four *AtFAD2* orthologs have been identified in total: the major-effect C01 and A05 QTL plus their homoeologous copies on chromosomes A01 and C05 (Yang et al., 2012). Although *BnFAD2.A01* appears to be a pseudogene, the three other copies are functional.

Variants in the *B. napus FAD2* copies have previously been found to affect oleic acid content. A single nucleotide polymorphism in the *BnFAD2.C01* coding region resulted in an increase in oleic acid content up to 77%, while for *BnFAD2.A05*, a single-nucleotide substitution (Hu et al., 2006) or a 4-bp insertion (Yang et al., 2012) in the coding region resulted in an increase in oleic acid content up to 75%. A new high oleic acid mutant reported by Long et al. (2018) showed two SNPs in *BnFAD2.A05* and *BnFAD2.C05*, again confirming the importance of *BnFAD2* for oleic acid content. Moreover, loss of function of *BnFAD2* in *B. napus* via gene knockout (Wells et al., 2014), RNA interference (Peng et al., 2010), and CRISPR/Cas9-mediated genome editing (Okuzaki et al., 2018), all result in an increase in oleic acid content of up to 84–85%. Although great success has been achieved by manipulating *BnFAD2* to obtain high oleic acid oilseed rape, most high oleic germplasm to date relies on mutation of *BnFAD2.A5*, and seems to be linked to poor agronomic performance of the plant, particularly at lower temperatures (Kinney, 1994; Bai et al., 2018). Development of novel genetic resources of oilseed rape with both high oleic acid content and superior field performance is thus highly desirable.

In the present study, QTL mapping was performed in *B. napus* using super-high (~85%) and normal-oleic acid (~65%) lines as parental lines. Two major QTLs were identified on A05 and C05, corresponded to the genes *BnFAD2.A05* and *BnFAD2.C05*. Two previously unreported mutant alleles were identified for each of these genes. Subsequently, we developed and validated functional KASP markers for these genetic variants. Our results enhance our knowledge of the role of allelic variation in determining high oleic acid content and will facilitate breeding for high oleic acid varieties in oilseed rape.

## Materials and Methods

### Plant Materials and Phenotypic Evaluation

**Figure 1:**
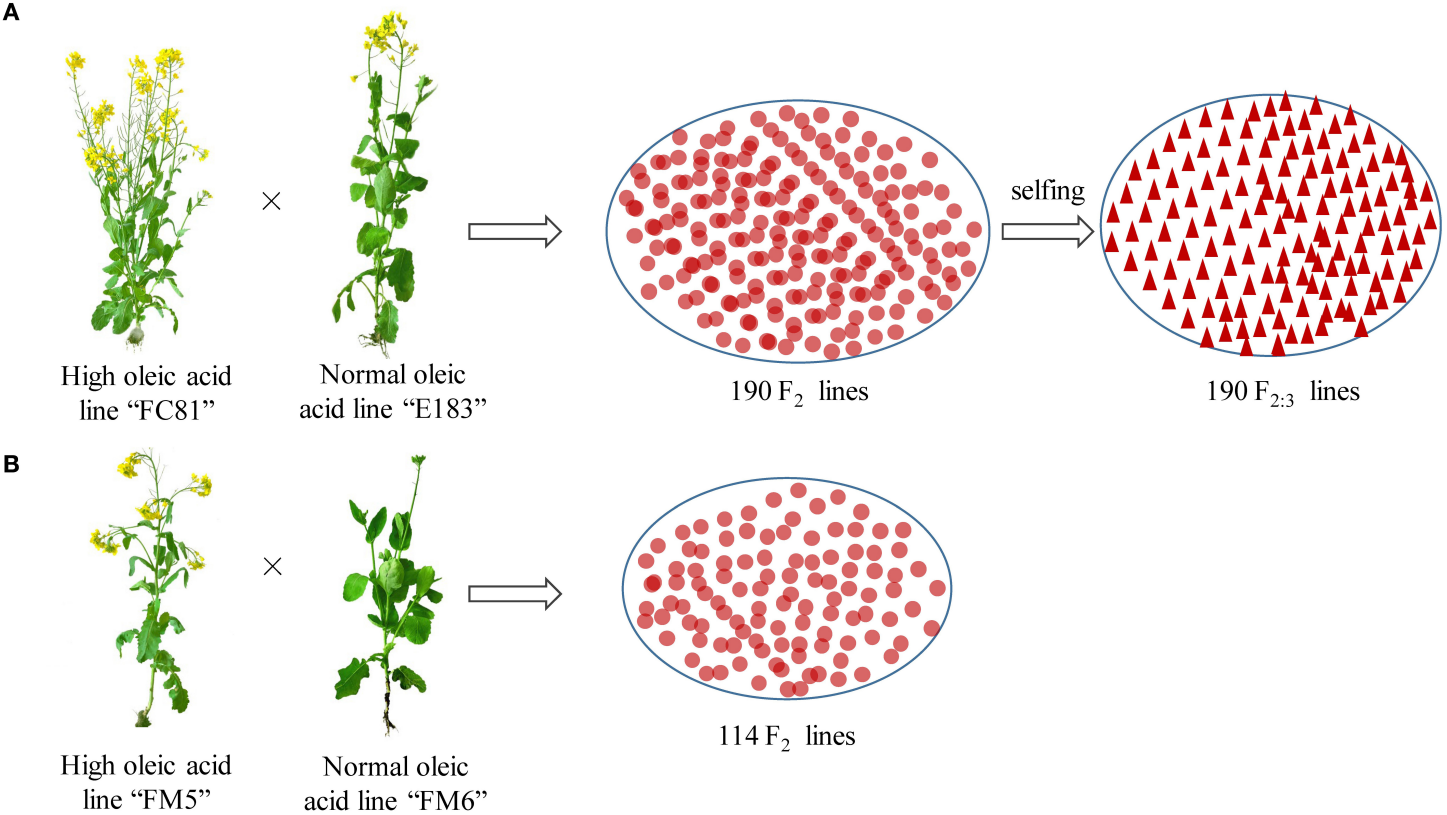
Experimental material and crossing design. **(A, B)** Represent the QTL mapping population and validation population, respectively.

A biparental population of 190 segregating F_2:3_ lines was derived from F_2_ offspring of a cross between a super-high (“FC81”, a homozygous M6 mutated line obtained from the ^60^Co-γ radiation with 85% seed oleic acid content) and normal-oleic acid (E183, 65%) lines, and was used for QTL mapping of oleic acid (Figure 1). The validation population was an F_2_ population also derived from crosses between super-high (FM5, a M6 mutated line which origin from the same high oleic acid mutant with “FC81,” 83%) and normal-oleic acid (FM6, 65%) lines, which was used for functional marker validation (Figure 1). Seed oleic acid contents of the F_2:3_ lines were measured on self-pollinated seed samples collected from field evaluation of the F_2:3_ population over 3 years. The inflorescences of 10 randomly-chosen plants in each plot were covered in pollen-proof bags at the onset of flowering to prevent cross-pollination. Self-pollinated seeds were collected in the bags at maturity for quality analysis. Seed oleic acid contents of the F_2_ validation populations were measured from the self-pollinated seeds from the F_2_ lines. All trials were grown at the agricultural field station of Zhejiang Academy of Agricultural Sciences (Hangzhou, China), with a plot size of 4.5 m^2^ (1.5 m plot size of rows per plot).

Measurements for seed oleic acid content on the self-pollinated seeds were obtained by gas–liquid chromatography (GC) analysis with the Model 6890 GC analyser (Agilent Technologies, Inc., Wilmington, DE), following protocol described by Thies (1971), the phenotypic data of the 190 F_2:3_ lines was shown in Supplementary Table 1.

### SNP Marker Analysis

The Illumina Infinium *Brassica* 60K SNP Array (Clarke et al., 2016) was used for genotyping 190 F_2_ lines and two parental lines (Supplementary Table 2). Total genomic DNA was extracted using DP321-03 DNA extraction kits (Tiangen, Beijing, China). SNP genotyping was performed in the National Key Laboratory of Crop Genetic Improvement, National Subcenter of Rapeseed Improvement in Wuhan, Huazhong Agricultural University, Wuhan, China, according to the Infinium HD Assay Ultra manual protocols. Illumina HiSCAN scanner was used for imaging the hybridized chips. GenomeStudio v2011 (Illumina, Inc.) genotyping software was used for allele calling. SNP markers were named using the SNP plus index numbers assigned by GenomeStudio from the chip information (Clarke et al., 2016), followed by chromosome number. SNP positions were obtained by BLAST search (e ≤ 1e−50) against the *B. napus* genome reference Darmor v4.1 (Chalhoub et al., 2014).

### Linkage Analysis and QTL Mapping

Genetic linkage groups were constructed using the packages MSTmap (Wu et al., 2008) and JoinMap 4.0 softwares (Van Ooijen and Voorrips, 2006). Polymorphic SNP markers were grouped firstly by LOD 5.0, and marker orders were determined based on pairwise recombination frequencies, using MSTmap. Then, by applying the mapping function of Kosambi (1943) and a minimum LOD score of 3.0 using Joinmap 4.0, the marker order and distance were recalculated and confirmed. Markers with zero recombination were assigned to the same bin. QTL Detection for seed oleic content was performed in the F_2:3_ population using the composite interval mapping (CIM) procedure of the WinQTL Cartographer 2.5 software (Wang et al., 2005). A 1,000-permutation test was performed to estimate the significance threshold of the test statistic for each QTL based upon a 5% experiment-wise error rate (Churchill and Doerge, 1994).

### Gene Sequencing and Development of KASP Markers

The standard molecular cloning procedure described by Sambrook and Russell (2001) was followed to isolate the genomic sequence of the *BnFAD2* genes in the two parents. The sequences were aligned among clones using the software VectorNTI (www.invitrogen.com/VectorNTI).

For each functional SNP of the *BnFAD2* genes, two allele-specific forward primers and one common reverse primer were designed using the Primer Premier 5.0 program according to the standard KASP guidelines (Singh et al., 1998), and were named KASP-421 for the SNP *BnaA.FAD2.a* and KASP-1073 for the SNP *BnaC.FAD2.a*. For KASP-421, the two allele-specific primers were added with the standard FAM (5′-gttggaatggtggcgtcgatg-3′) and HEX (5′-gtgttggaatggtggcgtcgata-3′) tails respectively at the 5′ end, with a common reverse primer as follows: 5′-ggacgacaccgtcggcctca-3′. For KASP-1073, the two allele-specific primers were added with the standard FAM (5′-ggtggttaaggcgatgtggag-3′) and HEX (5′-cggtggttaaggcgatgtggaa-3′) tails respectively at the 5′ end, with the common reverse primer as follows: 5′-ccggttccacatagatacactcctt-3′.

The polymorphism of the developed KASP markers were firstly validated in four sequenced lines. The primer assay mixture comprised 46 μL ddH_2_O, 30 μL common reverse primer (μM), and 12 μL of each allele-specific forward primer (μM). KASP assays were carried out in 96-well plate formats using a Roche LightCycler 480-II instrument (Roche Applied Sciences, Beijing, China), and set up using 8 μl reaction volumes: 2.5 μL of 60 ng/μL DNA template, 2.5 μL of 2 × KASP master mixture, 0.07 μL of primer assay mixture, and 2.93 μL of ddH_2_O. PCR cycling was carried out as 94°C for 15 mins, followed by 10 touchdown cycles (94°C for 20 s; touchdown at 61°C initially and decreasing by 0.6°C per cycle for 60 s), followed by 26 additional cycles of annealing (94°C for 20 s; 55°C for 60 s). Fluorescence was read by the Pherastar and analyzed using the BMGPHERAstar Software.

## Results

### Variation for Seed Oleic Acid Content

**Figure 2:**
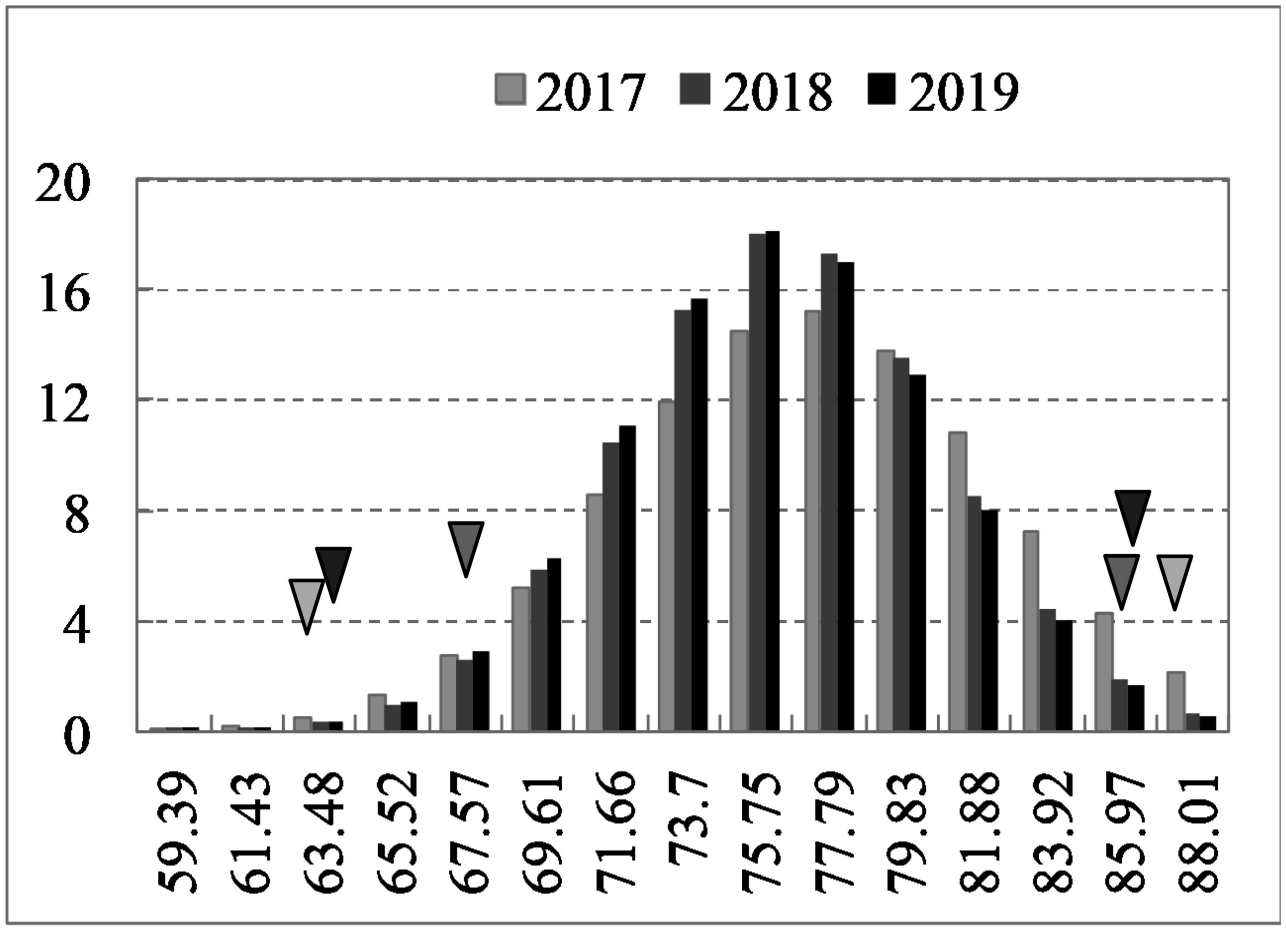
Frequency distributions of seed oleic acid content from 2017 to 2019. Triangles on the left and right of the figure represent the low oleic acid content line “E183” and high oleic acid content line “FC81”, respectively.

Over 3 years, the high oleic acid content parental line “FC81” exhibited 84.4–86.9% oleic acid in self-pollinated seeds from the field trials, while the normal-oleic acid parental line “E183” exhibited 62.7–66.2% oleic acid. Transgressive segregation of seed oleic acid content was detected in the F_2:3_ population, with normal distribution of values ranging from 59.4 to 87.6% (Figure 2). Significant differences between genotypes, but no environment-specific or genotype-by-environment interactions were found for oleic acid content (ANOVA, *P* < 0.01) (Supplementary Table 3). Similarly, relatively high heritability of oleic acid content was observed across environments (*H*^2^ = 72.9%). For the other fatty acid production, significant difference was observed for C18:2 content between low oleic acid content (<75%) and high oleic acid content (>75%) (*P* < 0.05), with 14.62 and 9.10% of C18:2 content between two groups, while no significant difference was observed for C18:0 and C18:3 between two groups, with 1.45 and 1.50% of C18:0 content, 6.08 and 5.07% of C18:3 content between two groups, respectively. These results suggested that no other fatty acid production changes except the C18:1 and C18:2 content.

### Linkage Mapping Identified QTL Controlling Oleic Acid Content

A total of 9728 SNP markers from the Brassica 60 K array showed expected segregation 1:1 ratio in the F2:3 population, which were used for genetic linkage construction. A set of 8128 SNP markers were successfully assigned to the 19 chromosomes of the A genome (A01–A10) and C genome (C01–C09) of *B. napus*, respectively. The genetic map spanned a genetic distance of 2311.16 cM, with an average distance of 0.28 cM between adjacent markers. As expected from previous genetic mapps of *B. napus* and the different size of chromosomes (Snowdon et al., 2002), the marker density and distribution considerably varied across chromosomes (Supplementary Figure 1). The highest marker density was found on chromosome A07, with 889 markers distributed over a genetic map distance of 75.53 cM. In the A genome four chromosomes showed gaps more than 20 cM, while only 1 chromosome in the C genome showed a gap over 20 cM.

The QTL analysis via CIM procedure in the software WinQTL Cartographer 2.5 revealed a total of four individual QTL for seed oleic acid content in individual environments, located across four chromosomes (A01, A05, A09 and C05) and each explaining between 3.00 and 52.09% of the phenotypic variation (Table 1). Of these, the QTL on A05 and C05 were major QTL, explained 45.02–52.09% and 14.74–44.29% of phenotype variations, respectively. The QTL on A05, A09 and C05 overlapped across environments, suggesting the reliability of these QTL across year environments. Negative additive effects were detected for all of these four QTL, indicating that the parent FC81 contributed to a strong increase in seed oleic acid content.

**Table 1:** QTL for seed oleic acid content in a segregating *Brassica napus* F_2:3_ population.

**QTL**	**Chr.^a^**	**Env.^b^**	**Add.^c^**	**QTL mapping**	
				**Pos.^d^**	***R*^**2**^ (%)^e^**
qOC17A01	A01	2017	−1.40	45.2–47.6	3
qOC17A05	A05	2017	−5.11	107.5–124.8	45.02
qOC17A09	A09	2017	−1.49	20.1–28.9	3.85
qOC17C05	C05	2017	−5.05	18.3–27.3	44.29
qOC18A05	A05	2018	−4.54	107.5–135.7	52.09
qOC18A09	A09	2018	−1.41	18.7–29.2	5.03
qOC18C05	C05	2018	−2.42	12.9–24.3	15.49
qOC19A05	A05	2019	−4.19	107.5–125.3	46.53
qOC19C05	C05	2019	−2.32	12.9–27.8	14.74

a *Chromosome*.

b *Field trials in the different years*.

c *Additive effect. The direction of additive effect is from the allele of “E183,” while a negative additive effect indicates an allelic contribution from “FC81”*.

d *Length of 2-LOD score confidence interval*.

e *Percentage of the phenotypic variance explained by each QTL*.

### Identification of Mutations in the *BnFAD2* Genes of the High Oleic Acid Line

**Figure 3:**
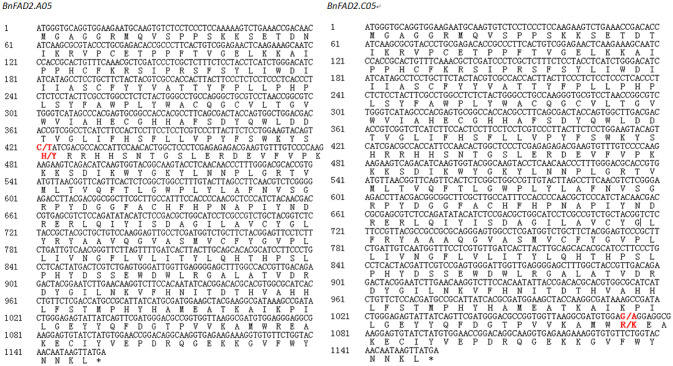
Nucleotide and protein sequence of *BnFAD2.A05* and *BnFAD2.C05* containing single nucleotide polymorphisms (SNPs) resulting in high oleic acid content in *Brassica napus*. Red bold font represented the positions of the SNP sites, marked as “wild type/mutant”.

By comparing of the QTL region with the reference genome of *B. napus*, we found that the two *BnFAD2* genes on A05 and C05, with key roles in controlling the oleic acid content, were physically anchored within the QTL intervals on A05 and C05 of the *B. napus* reference genome. Gene sequence comparison showed that all four cloned *BnFAD2* gene sequences from the two parental lines exhibited more than 95% sequence similarity to the corresponding gene sequences from the reference genome. Two SNPs across the entire coding region, C421T in the *BnFAD2.A05* and G1073A in the *BnFAD2.C05*, were detected by comparing the two parental lines (Figure 3), and resulted in the amino acid changes H141Y and R358K, respectively.

### KASP Marker Development and Functional Confirmation

**Figure 4:**
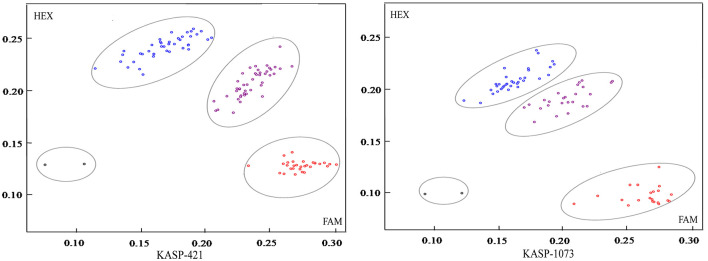
KASP marker genotyping in a segregating *Brassica napus* F_2_ population using functional markers KASP421 and KASP 1073 for high oleic acid content. The red circle represents homozygous alleles derived from parent line “E183” with normal oleic acid content, the blue circle represents homozygous alleles derived from parent line “FC81” with high oleic acid content, and the purple circle represents heterozygous loci. The black circle represents negative control.

**Figure 5:**
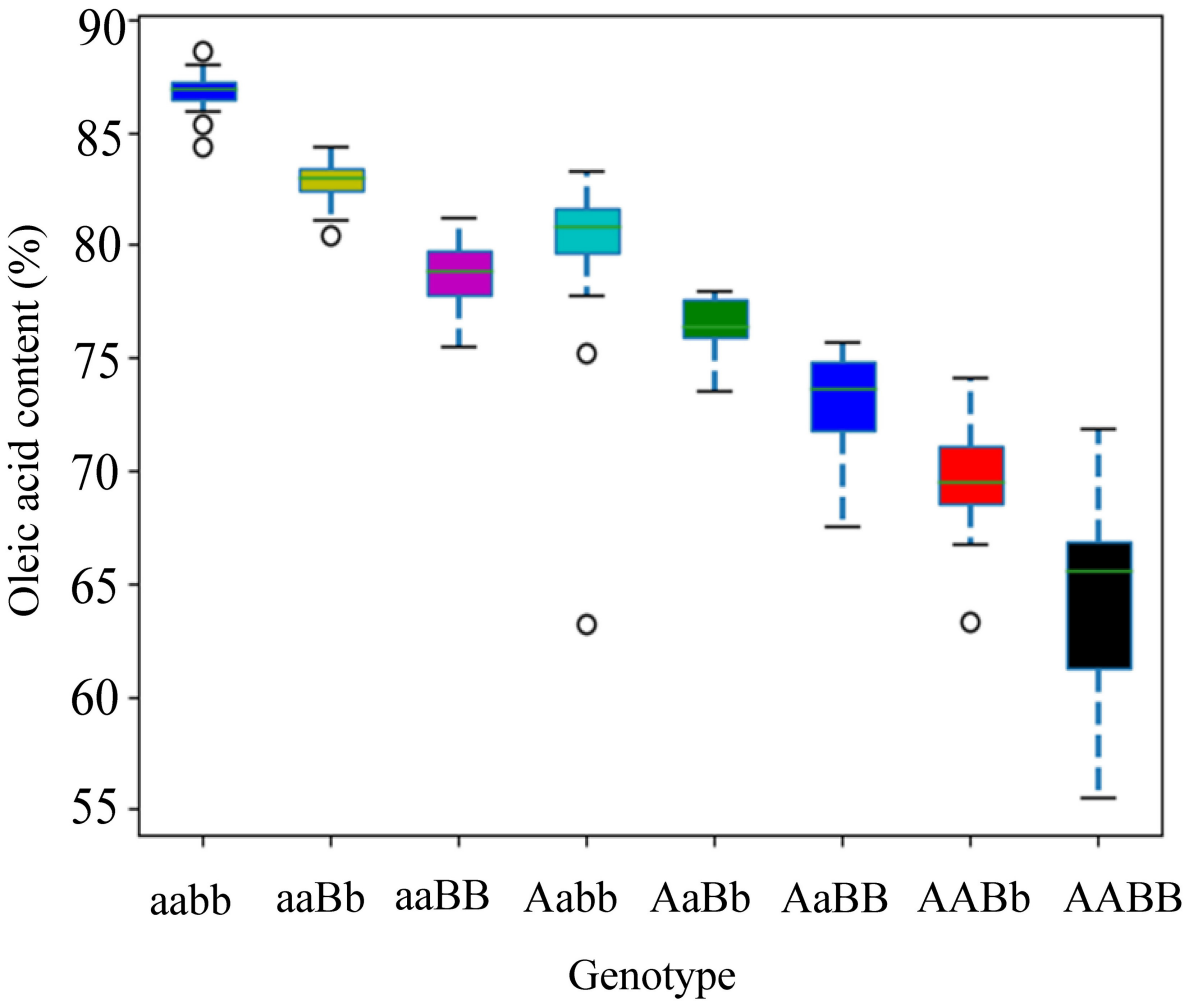
Seed oleic acid content of different *BnFAD2* genotypes distinguished by allelic KASP primers KASP421 and KASP 1073 in an F_2_ population. The “A” and “B” represents wild type alleles by allelic KASP primers KASP421 and KASP 1073, respectively, while “a” and “b” represents mutated alleles by allelic KASP primers KASP421 and KASP 1073, respectively.

Allelic KASP primers were designed to detect the two *BnFAD2* SNPs (C421T and G1073A). Both the KASP421 primer pair (specific to C421T in the *BnFAD2.A05*) and the KASP1073 primer pair (specific to G1073A in the *BnFAD2.C05*) showed perfect segregation between the two parental lines as functional markers. In order to test the phenotypic prediction effect of the two KASP markers on seed oleic acid content, we genotyped a new segregation F_2_ population containing 114 lines using both markers (Figure 4). For the marker KASP421, the seed oleic acid content in the F_2_ group with the allele from “FC81” averaged 83.6%, significantly higher than that of “E183” (*P* < 0.01), with average oleic acid content of 68.5%. For the marker KASP1073, the seed oleic acid content in the F_2_ group with the allele from “FC81” showed average oleic acid content of 82.0%, significantly higher than that of “E183” (*P* < 0.01) with average oleic acid content of 70.7%. The lines containing both alleles from “FC81” showed significantly higher oleic acid content than lines containing both alleles from “E183” or lines containing one or the other allele (*P* < 0.01) (Figure 5).

## Discussion

In the previous studies, most high oleic germplasm (>80%) seems to be linked to poor agronomic performance of the plant (Kinney, 1994; Bai et al., 2018). However, the high oleic lines in our populations did not show obvious negative effect on agronomic traits such as yield, flowering time, and oil content, which provided excellent germplasm for high oleic acid breeding in rapeseed. Based on this material, we identified four QTL explaining oleic acid content, two of which (A05 and C5) turned out to be due to novel variants of the well-characterized *Brassica FAD2* genes, which convert oleic into linoleic acid, and two of which (A01 and A09) had unknown causal genes. Of these, the QTL on A05 and C05 were major QTL, explained 45.02–52.09% and 14.74–44.29% of phenotype variations, respectively. The QTL on A01 and A09 were minor QTL, explained phenotype variations <10%. The total phenotype contributions of QTL on A05 and C05 in 3 years were 89.31, 67.58 and 61.27%. Thus, the QTL on A05 and C05 will be the key loci for genetic improvement of high oleic acid breeding in rapeseed.

A number of studies have previously undertaken to map the genetic factors responsible for oleic acid content in rapeseed via either linkage mapping studies or by association approaches (Burns et al., 2003; Hu et al., 2006; Zhao et al., 2008, 2019; Smooker et al., 2011; Yan et al., 2011; Yang et al., 2012; Wen et al., 2015; Niklas et al., 2016; Qu et al., 2017; Bao et al., 2018; Chen et al., 2018). Of the QTL we identified, the major QTL on A05 (*BnFAD2*) has been repeatedly identified across previous studies (Hu et al., 2006; Yang et al., 2012). A minor QTL on A01/C1 has also been detected previously for seed oleic acid content (Hu et al., 2006), but we are unable to confirm if this is the same one that we found due to the lack of common flanking markers between studies and the unknown physical location of the markers from the earlier study. A minor QTL on A09 controlling oleic acid content was reported and fine-mapped by Zhao et al. (2019). However, the physical location of this QTL was at 28.00–28.07 Mb on chromosome A09, while our A09 QTL was located at 30.47–32.86 Mb. As both studies used the same SNP array and reference genome, it seems this could possibly indicate two different QTL. Although QTL for oleic acid content on chromosome C05 have not previously been identified, the direct mutation of the underlying *BnFAD2-2* gene has been positively associated with elevated oleic acid levels (Long et al., 2018). Comparative to previous work, most of the QTL we identified appear to play a major role in determining oleic acid content across other populations and environments, indicative of a common genetic control. The QTL we identified on chromosome A09 (30.47–32.86 Mb) appears to be a novel locus controlling seed oleic acid in oilseed rape, and could be more genotype- or environment-specific.

The *Bnfad2.A05* and *Bnfad2.C05* alleles in this study were novel relative to previously identified variants (Table 2). However, beneficial fatty acids facilitate the utilization of rapeseed oil (Napier et al., 2014), and the relationship between *BnFAD2* mutations and oleic acid content in rapeseed seeds is well known (Hu et al., 2006; Yang et al., 2012; Long et al., 2018); hence, several high oleic acid rapeseed lines have previously been created by mutagenesis, almost all via mutation of *FAD2* genes (Auld et al., 1992; Schierholt et al., 2001; Hu et al., 2006; Spasibionek, 2006; Yang et al., 2012). *BnFAD2* is a trans-membrane protein with three conserved histidine-rich motifs (also called histidine boxes, H-boxes), which form the active center of the enzyme (Shanklin et al., 1994). These three H-boxes are 105-HECGHHAF-111, 137-WKYSHRRHH-145, and 315-HVAHHLFS-323 (Tanhuanpää et al., 1998). Mutations in or near the H-boxes have a higher probability of inhibiting BnFAD2 enzyme activity. Although confirmation of the effects of different mutated positions requires additional functional analyses, for breeding purposes artificial mutation in the active H-box centers should be the most efficient for elevating seed oleic acid levels. In our study, the mutation *Bnfad2.A05* was H141Y, which was located within the second H-box. The mutation site of *Bnfad2-C05* was R358K, near the third H-box motif. Therefore, it is not surprising that these two alleles resulted in super high seed oleic acid content. Additionally, in soybean, Ser-185 just outside the *BnFAD2* H-boxes was validated to play a key role in regulating post-translational modifications that directly affected FAD enzyme activity (Tang et al., 2005). Future work should further explore the relationship between mutation sites and oleic acid content in *B. napus*.

**Table 2:** The mutation of *BnFAD2* genes and corresponding oleic acid content in *B. napus*.

**Gene**	**Mutation position**	**Mutation**	**Oleic acid content**	**Mutation type**	**Reference**
*BnaFAD2-A05*	421 bp	C → T	88.57%	Physical mutagenesis	The present study
*BnaFAD2-C05*	1073 bp	G → A			
*BnaFAD2-A05*	567 bp	insertion	77.20%	Chemical mutagenesis	Yang et al., 2012
*BnaFAD2-A05*	59 bp	A → C	71%	Physical mutagenesis	Zhang et al., 2008
*BnaFAD2-C05*	722 bp	A → T			
	614 bp	A → G			
*BnaFAD2-C05*	270 bp	G → A	91.50%	Physical mutagenesis	Guan et al., 2006
*BnaFAD2-A05*	316 bp	G → A	85%	Physical mutagenesis	Long et al., 2018
*BnaFAD2-C05*	908 bp	G → A			
*BnaFAD2-A05*	553 bp	C → T	75%	Chemical mutagenesis	Hu et al., 2006
*BnaFAD2-C05*	36 mutations occurred at different positions	Substitution	69.4–86.4%	Chemical mutagenesis	Wells et al., 2014
*BnaFAD2-C05*	39 mutations occurred at different positions	Substitution	63.21–87.26%	Chemical mutagenesis	Bai et al., 2018
*BnaFAD2-A05*					
*BnaFAD2-C01*					
*BnaFAD2-A05*	18 mutations occurred at different positions	Deletion and insertion	71.7–80%	CRISPR/Cas9	Okuzaki et al., 2018

One of the goals of this research was to develop genetic markers for high oleic acid breeding. Clearly distinguishing the homologous from homoeologous genotypes was a key factor. As the *Brassica* A and C genome *FAD2* copies have very high sequence similarity (Lee et al., 2013), it is difficult to design specific primers to amplify the mutant SNP sites in *BnFAD2* genes. In this study, we successfully developed SNP-based KASP markers corresponding to each SNP site and clearly genotyped lines in a segregating population. The developed KASP markers were stable and can unambiguously differentiate the parent and hybrid genotypes, facilitating marker-assisted selection for the high oleic acid content trait. Given that the *Bnfad2.A05* and *Bnfad2.C05* alleles are located on different chromosomes, no linkage effects exist between these genes. Moreover, additive genetic effects contributed much more to oleic acid content than dominance and epistasis effects: as additive effects are the most highly heritable, high oleic acid content can therefore be easily transferred in breeding. The developed KASP markers are predicted to facilitate breeding for high oleic acid rapeseed varieties.

## Data Availability Statement

The original contributions presented in the study are included in the article/Supplementary Material, further inquiries can be directed to the corresponding author/s.

## Author Contributions

YF conducted the whole experiment and wrote the manuscript, AM assisted with interpretation of results, manuscript writing and revision, YZ designed primers and participated in the field experiment and seed quality analysis, HY directed the project and contributed to the writing. All authors contributed to the article and approved the submitted version.

## Conflict of Interest

The authors declare that the research was conducted in the absence of any commercial or financial relationships that could be construed as a potential conflict of interest.

## Publisher's Note

All claims expressed in this article are solely those of the authors and do not necessarily represent those of their affiliated organizations, or those of the publisher, the editors and the reviewers. Any product that may be evaluated in this article, or claim that may be made by its manufacturer, is not guaranteed or endorsed by the publisher.
