# Supplementary material for: Identification and Development of KASP Markers for Novel Mutant BnFAD2 Alleles Associated With Elevated Oleic Acid in Brassica napus
Source: Front Plant Sci. 2021 Jul 26;12:715633. doi: 10.3389/fpls.2021.715633 (PMC8350730; doi:10.3389/fpls.2021.715633)
Supplement: Supplementary file 2 [file Data_Sheet_1.DOC]

Identification and Development of **KASP Markers** for Novel Mutant *BnFAD2* Alleles Associated with Elevated Oleic Acid in *Brassica napus*

Ying Fu**^1^, Annaliese S. Mason^2^, Yaofeng Zhang^1^ and Huasheng Yu**^*^**^1^**

^1^Institute of Crop and Nuclear Technology Utilization, Zhejiang Academy of Agricultural Sciences, Hangzhou, China

^2^Plant Breeding Department, The University of Bonn, Katzenburgweg 5, 53115 Bonn, Germany

*Corresponding author

Phone: +86 (0) 571 86404096; Fax: +86 (0) 571 86404096; E-mail: yuhuasheng-0@163.com

Email addresses for all authors: [fy97@163.com](mailto:fy97@163.com); [annaliese.mason@uni-bonn.de](mailto:annaliese.mason@uni-bonn.de); [y.f.zhang@163.com](mailto:y.f.zhang@163.com); yuhuasheng-0@163.com

Figure S1
